# An accurate formula to calculate exclusion power of marker sets in parentage assignment

**DOI:** 10.1186/1297-9686-44-36

**Published:** 2012-12-03

**Authors:** Marc Vandeputte

**Affiliations:** 1 INRA, UMR1313 Animal Genetics and Integrative Biology, Jouy-en-Josas, 78350, France; 2 Ifremer, UMR110 INTREPID, Palavas-les-Flots, 34250, France

## Abstract

In studies on parentage assignment with both parents unknown, the exclusion power of a marker set is generally computed under the hypothesis that the potential families tested are independent and unrelated samples. This tends to produce overly optimistic exclusion power estimates. In this work, we have developed a new formula that gives almost unbiased results at the population level.

## Findings

Parentage assignment using genomic markers, usually microsatellites, is now widely used for research on population ecology and evolution [1], as well as in selective breeding, particularly for aquatic species. Indeed, maintaining pedigrees for these species is a challenge because of the very small size of individuals at hatching, which prevents physical tagging [2]. When developing a marker set for parentage assignment, it is important to be able to predict the assignment efficiency from *a priori* data. Exclusion probabilities are easily calculated from allele frequencies and are commonly used to quantify the efficiency of individual markers for parentage assignment. The most frequently used exclusion probability is the probability to exclude a random parent pair that is unrelated to the individual tested (named *Q*_*3*_ in [3], here *Q*_*3i*_ for each locus *i*). Since a single locus is generally not sufficient to exclude all potential parent pairs, several loci have to be combined to reach an appropriate combined exclusion probability *Q*_*3*_, which is calculated as the product of the individual non-exclusion probabilities of all *L* loci:

(1) $$Q_{3}=1-\overset{L}{\underset{i=1}{\prod }}\left(1-Q_{3i}\right)$$

Then, the combined exclusion probability is raised to the power of the total number of potential parental pairs to be excluded. With *N* possible parent pairs (including the correct one), this number is *N*-1, and the probability to have all parent pairs excluded except the correct one is the theoretical probability of having a unique assignment [4,5]:

(2) $$P_{u}={Q_{3}}^{N-1}$$

However, experience shows that the predicted assignment rates using this formula are often too optimistic, especially in factorial designs, i.e. when the mating structure is unknown and thus all possible mother-father combinations must be taken into consideration [4,6]. It is then necessary to make two assumptions when applying formulae (1) and (2), i.e. (i) exclusion of the *N*-1 incorrect parent pairs represents *N*-1 independent tests and (ii) all excluded parents are unrelated to the offspring, which justifies the use of probability *Q*_*3*_. However, in practice, these assumptions are never met. While the lack of independence between tests does not prevent formula (2) to yield good approximations [5], the second problem is generally overlooked.

**Figure 1:**
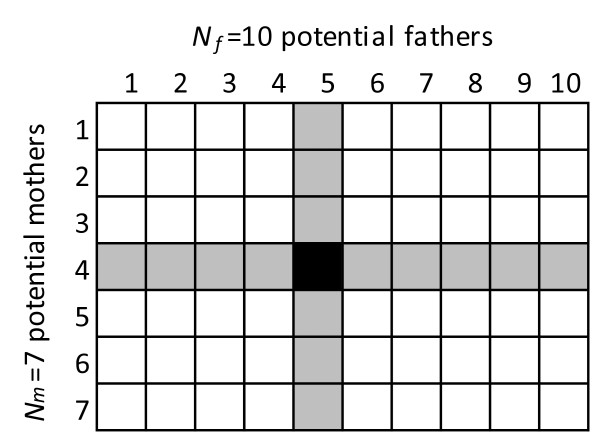
**Types of family relationships to be excluded for an offspring.** Types of family relationships to be excluded for an offspring with *N*_*m*_ potential mothers and *N*_*f*_ potential fathers; black = true family of an offspring; grey = *N*_*f*_ -1 families that share the same mother and *N*_*m*_ -1 families that share the same father, that have to be excluded; white = (*N*_*m*_ -1)(*N*_*f*_ -1) pairs of parents that are unrelated to the true parents and that also have to be excluded.

The most commonly encountered situation is when offspring are collected from a population that has a number of potential parents. The mating structure may be known (in some farmed populations) or not (in the wild or in farmed populations where parents are allowed to mate “naturally”). The practical aim of such studies is to identify the true parent pair of every genotyped offspring that derives from the sampled parents, which means excluding all parent pairs except the true one. Except in very specific cases where only single pair matings occur according to a perfectly known mating structure, the sole use of *Q*_*3*_ is disqualified because some potential half-sib families will have to be excluded. This is especially true when no mating structure is assumed (all mother-father combinations are considered possible, as in Figure 1) and thus the half-sib families cannot be considered to be unrelated to the correct family under consideration. The general approach is to exclude all mother-father combinations other than the true one, without taking a mating structure into account since, in most cases, the aim is to establish or check the mating structure.

Another exclusion probability *Q*_*1*_ was initially proposed by Jamieson [7] to calculate the probability to exclude one parent when the other parent is known, which is relevant to the exclusion of parents from half-sib families sharing one parent with the correct family. Probabilities *Q*_*1i*_ and *Q*_*3i*_ can be calculated for each locus with the following formulae [3]:

(3) $$Q_{1i}=1-2S_{2}+S_{3}+2S_{4}-2S_{2}^{2}-3S_{5}+3S_{3}S_{2}$$

(4) $$Q_{3i}=1+4S_{4}-4S_{5}-3S_{6}-8S_{2}^{2}+2S_{3}^{2}+8S_{3}S_{2}$$

with $S_{t}=\underset{j}{\sum }p_{j}^{t}$ and *p*_*j*_ the frequency of the *j*^*th*^ allele of locus *i* in the population. Combined probabilities over all loci, *Q*_*1*_ and *Q*_*3*_ can be calculated with formula (1).

Then, the combined probability of having a unique assignment among parent pairs that share one parent with the true parental pair is:

(5) $$P_{u1}=Q_{1}^{N_{f}-1}Q_{1}^{N_{m}-1}=Q_{1}^{N_{f}+N_{m}-2}$$

while the probability of having a unique assignment among unrelated parent pairs is:

(6) $$P_{u3}=Q_{3}^{\left(N_{f}-1\right)\left(N_{m}-1\right)}$$

Since the probability of having a unique assignment requires having both unique assignments within related pairs and within unrelated pairs, the global probability of having a unique assignment (also named exclusion power) is:

(7) $$P_{u}=Q_{1}^{N_{f}+N_{m}-2}Q_{3}^{\left(N_{f}-1\right)\left(N_{m}-1\right)}$$

It is then clear that the probability of having a unique assignment decreases exponentially as the number of potential parents increases, as already underlined by Wang [[5]]. However, the rate of decrease depends on whether term *Q*_*1*_ or term *Q*_*3*_ in formula (7) is most influential. Dodds et al. [3] have already shown that *Q*_*3i*_ is always greater than *Q*_*1i*_ for a given locus regardless of the allelic frequencies [3].

In the work reported here, we studied the relative importance of *Q*_*1*_ and *Q*_*3*_ using idealized loci, with three, five or eight equally frequent alleles. Individual *Q*_*1i*_ values were 0.370 for a locus with three alleles, 0.595 for a locus with five alleles and 0.743 for a locus with eight alleles, while the values for *Q*_*3i*_ were 0.519 for a locus with three alleles, 0.772 with five alleles and 0.898 with eight alleles. In most cases, these values reflect microsatellites with low, moderate or high variability.

**Figure 2:**
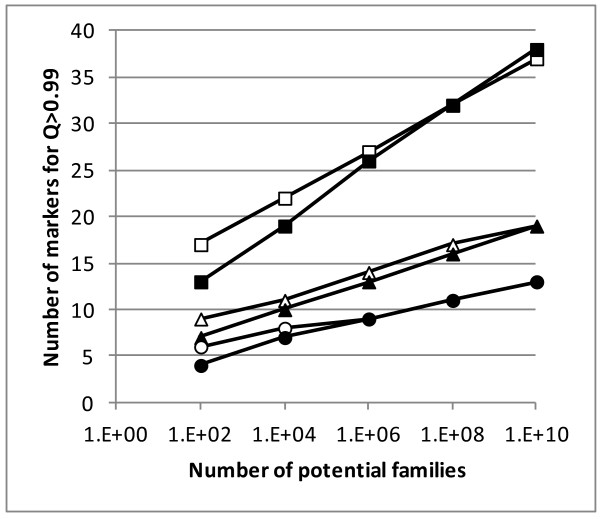
**Number of markers to achieve exclusion power greater than 0.99 for both terms in formula (7).** Number of markers to achieve exclusion power greater than 0.99 for both terms in formula (7); white symbols for the *Q*_*1*_ term; black symbols for the *Q*_*3*_ term; squares = loci with three equally frequent alleles; triangles = loci with five equally frequent alleles; circles = loci with eight equally frequent alleles; the situations simulated included *N* potential fathers and *N* potential mothers and, thus, *N*^*2*^ families.

As shown in Figure 2, in general a larger number of loci were needed for the *Q*_*1*_ term to exceed 0.99 compared to the *Q*_*3*_ term, except for very high numbers of potential families (≥ 10^6^ for loci with eight alleles, ≥ 10^10^ for loci with five alleles and ≥ 10^8^ for loci with three alleles). The only case in which the *Q*_*3*_ term required more loci than the *Q*_*1*_ term to reach 0.99 was with tri-allelic (low variability) loci and more than 10^10^ potential families. Thus in most cases, and especially when the number of potential families is moderate and the variability of the markers is low or intermediate, *P*_*u*_ will be governed by the *Q*_*1*_ term, contrary to the general view [4].

One important thing to note is that formula (7) does not assume a mating structure. This is because no mother-father combination is excluded *a priori* on the basis of pre-existing knowledge about mating structure and, thus, exclusion is performed on the basis of a full factorial design (Figure 1), which is the general case when no mating structure is assumed. It may be possible to consider fewer combinations when the mating structure is known and thus, modify the exponents of *Q*_*1*_ and *Q*_*3*_ in formula (7), but this approach is not recommended since it limits the generality of the estimated assignment power.

When comparing our results with those previously reported in the literature [4,6], we found that, except for marker sets with a very low assignment power, formula (7) gives much more accurate results than formula (2) (Table 1). When assignment power is low, formula (7) tends to underestimate it, making it a conservative estimate. Other problems (linkage between markers, genotyping errors, inbreeding, use of relatives as parents, sampling errors, etc.) may further decrease the assignment power of a marker set but the systematic gap between the assignment power computed with formula (2) and the theoretical one (up to now approached only by simulation) is the main cause of overestimation of the power of marker sets for parentage assignment [6]. Since formula (7) is easily computed based on allele frequencies in a spreadsheet, we recommend its use to design marker sets with an appropriate exclusion power.

**Table 1:** Comparison of predicted and simulated exclusion power *P*_*u *_of idealized and real marker sets

				**Exclusion power *P***_***u***_		
**Type of markers**	**Size of factorial design** (***N***_***f ***_**x *N***_***m***_)	**Alleles/ locus**^**a**^	**Number of loci**	**Predicted** (**Eq.**2)*	**Simulated**	**Predicted** (**Eq.**7)**
Idealized markers (equally frequent alleles)	10x10	5	3	0.3064	0.2123	0.1104
	10x10	5	6	0.9861	0.9163	0.9131
	10x10	5	9	0.9998	0.9947	0.9946
	10x10	5	12	1.0000	0.9997	0.9996
	10x10	10	3	0.9690	0.8448	0.8334
	10x10	10	6	1.0000	0.9987	0.9986
	20x20	5	3	0.0085	0.0321	0.0001
	20x20	5	6	0.9453	0.8143	0.8037
	20x20	5	9	0.9993	0.9884	0.9884
	20x20	5	12	1.0000	0.9993	0.9993
	20x20	10	3	0.8810	0.6717	0.6409
	20x20	10	6	1.0000	0.9972	0.9971
Real microsatellites	76x13	20.1	8	1.0000	0.9994	0.9993
	75x26	21.7	6	0.9999	0.9934	0.9934
	41x8	19.3	6	0.9999	0.9928	0.9920
	20x2	16.3	4	0.9986	0.9465	0.9421
	147x8	7.5	8	0.9911	0.8604	0.8636
	96x8	7.6	8	0.9975	0.9473	0.9422
	24x10	7.8	8	0.9990	0.9712	0.9782
	100x101	7.5	12	0.9968	0.9708	0.9696

Predicted and simulated values from Villanueva et al. [4] for idealized marker sets and from Vandeputte et al. [6] for real marker sets; simulated values were obtained for 800 offspring per cross in [4] and 1000 offspring per cross in 100 independent parent samples in [6]; both used *formula (2) to calculate predicted values, which were compared to the values obtained with **formula (7) described here; ^a^for real loci, average number of alleles per locus.

## Competing interests

The author declares no competing interests.

## Authors’ contributions

MV identified the question, established the formula, tested it on real data and wrote the article.

## Author information

MV works in fish quantitative genetics at the INRA-Ifremer research group on sustainable fish breeding. One of the main tools used for fish quantitative genetics studies is parentage assignment with microsatellite markers, which he contributes to optimize.
